# Activating urban infrastructure for health: A hope framework for health and planetary resilience

**DOI:** 10.1371/journal.pgph.0006183

**Published:** 2026-04-24

**Authors:** Tolu Oni

**Affiliations:** Cambridge Africa Diaspora Centre for Healthy Hopeful Urban Futures, Institute of Metabolic Science (IMS) Epidemiology, University of Cambridge, Cambridge, United Kingdom; PLOS: Public Library of Science, UNITED STATES OF AMERICA

Climate change and health risks are converging in cities, exposing structural weaknesses in existing institutions. Nowhere is this more pressing than in rapidly urbanising contexts particularly on the African continent [1] where more than 60 percent of urban infrastructure investments to be made by 2050 are pending [2]. Such contexts have a transformative opportunity to build cities that are healthy by design. Yet, too many are expanding without health-centred design like integrated active transport networks and accessible green space that improve air quality, support safe physical activity and reduce disease burden.

Changing course to secure planetary health in an urbanising world will require more than technical solutions. It demands a reimagining of how cities are designed and governed, clarity about *what* must change and a fundamental shift in *how* that change is pursued. As argued here, it also requires cultivating hope – not as a sentiment, but as an institutional capacity that enables healthier urban futures to be realised.

## Hope as an activating force for urban health

Hope is often dismissed as naïveté, a passive feeling that things might improve. This obscures its deeper potential as an active orientation that combines desire for a particular future, belief in its plausibility, and agency to act toward it [3]. Hope can function as a health-enabling capacity as evidenced by studies showing better health outcomes in individuals with higher hope scores [3], and a dose-response relationship with moderately and highly hopeless men at increased risk of all-cause and cause-specific mortality relative to men with low hopelessness scores [4].

Beyond the individual, collectivist societal contexts frame hope as communal and intergenerational, and rooted in shared memory [5], with the potential to activate collective agency and civic engagement. When applied to how institutions are designed and complexity is governed, this potential can mobilise collective capacity and influence urban decision-making in ways that promote healthier environments with long-term stewardship of place.

## Operationalising hope to activate urban infrastructure as health infrastructure

More than 80 percent of the factors that shape health lie outside healthcare [6]. Yet the dominant model of health delivery is in healthcare, largely structured around disease treatment and biomedical intervention. While the World Health Organization’s health systems framework [7] is essential, its building blocks are not designed to ensure upstream determinants – the environments in which people live, work and move – systematically generate health. The design of cities influences the quality of the air residents breathe, the safety and feasibility of active transport, patterns of social cohesion and the accessibility of nutritious food but these environments are rarely designed explicitly to optimise health. In this sense, urban infrastructure is health infrastructure, whether it actually serves this purpose or not.

This piece’s proposition is that hope provides a structured approach to activating urban infrastructure as health infrastructure.

In this framing, hope infrastructure refers to intentionally designed systems and processes embedded within urban governance, education and civic institutions that cultivate agency, participation and anticipatory capacity. This positions hope as both a method, cultivated through inclusive processes that embed futures orientation into how cities plan, design and govern for health, and as a normative public value for institutions, on par with resilience or sustainability.

**Fig 1:**
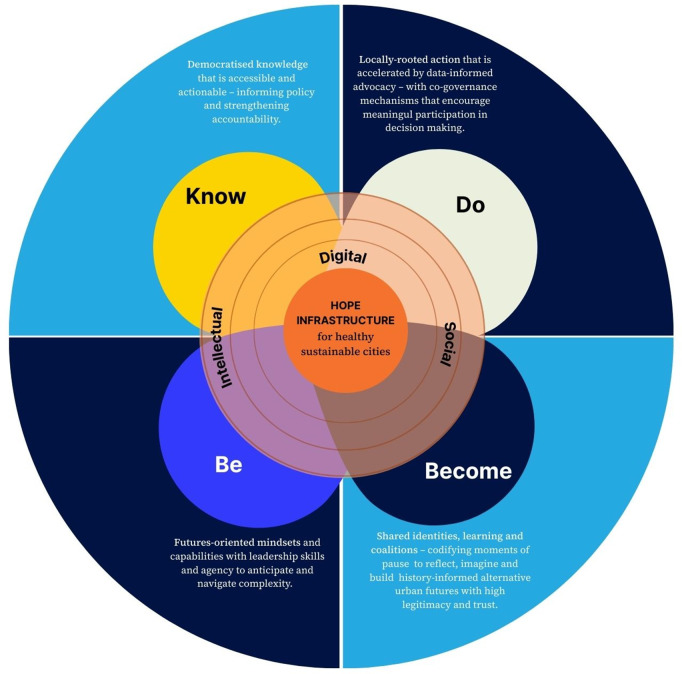
Hope as Health-activating Infrastructure: “Know-Be-Do-Become” framework.

Treating hope as infrastructure therefore shifts it from an individual disposition to a systemic capacity and a four-dimensional Know–Be–Do–Become framework is offered to operationalise this proposition. (Fig 1).

- **Know: Democratised, action-oriented knowledge.**
  - Citizen science, open data and participatory research enable city residents to surface local health and climate risks, from air pollution along transport corridors to unsafe pedestrian infrastructure and food deserts. By aligning scientific evidence with lived urban experience, these approaches make invisible exposures visible to urban planners and decision makers, thereby informing urban design.
- **Do: Connecting knowledge to action.**
  - Community networks, civic platforms and co-governance mechanisms translate locally generated evidence into advocacy and targeted urban interventions. When linked to municipal processes, transport policy, zoning decisions and budget allocations, local action strengthens civic agency (and vice versa) and demonstrates how resident participation can influence the design of urban systems to improve health outcomes.
- **Be: Internal capabilities for leadership and foresight.**
  - Futures literacy, systems thinking and reflexivity equip individuals, particularly youth, with the capacity to understand how infrastructure and governance shape city life over time and to play an active role in shaping healthier urban futures. Hope is not inherently benevolent. Without reflexivity, it can reproduce narrow worldviews, exclusionary visions of progress, or planning practices that deepen spatial inequities. To hope well requires understanding how history, power, marginalisation and inequitable investments shape what futures are imagined and prioritised, and for whom health is created.
- **Become: Collective learning and transformation.**
  - Cross-city exchange, intergenerational dialogue and reflective practice enable coalitions of municipal authorities, city leaders, community organisations and youth networks to learn collectively and evolve governance practices. These processes can support adaptive governance, strengthen solidarity across neighbourhoods and cities and counter short-term political cycles that often undermine long-term urban health and climate resilience.

## Hope infrastructure in action

Ultimately operationalising hope requires confronting questions of power. Traditional institutions concentrate authority and expertise, reinforcing hierarchies that often exclude those most affected by health risks. In contrast, institutionalising hope requires reframing power not as a finite resource to be hoarded, but as something generative that circulates through relationships, participation and shared purpose [8]. When embedded in governance, education and civic processes, hope infrastructure seeks to redistribute agency and expand who can shape urban health futures.

Elements of the hope as infrastructure framework articulated are emerging. In Nigeria, the civic tech initiative BudgIT [9] simplifies public finance data, enabling citizens to track government spending and advocate for health-relevant investments such as sanitation and transport – strengthening transparency, accountability and trust while connecting knowledge to action (Know, Do). Similarly, the UrbanBetter Cityzens initiative [10] uses a “precision advocacy” model, with shared digital platforms and cross-city learning networks, linking physical activity and environmental citizen science to equip young people to measure air quality or walkability and engage decision-makers to inform urban policy (Know, Be, Do, Become). While promising, the contribution of such initiatives to population health depends on their integration with institutional ecosystems and urban governance processes.

## Conclusion

In rapidly urbanising contexts where health risks and opportunities are most acute, cities require more than improved infrastructure. They require the capacity to activate that infrastructure for health.

Hope, as a structured practice, is an activating force that can enable urban infrastructure to function as health infrastructure. Streets, transport systems, housing and public space already shape health outcomes. Whether they generate health or reproduce inequity depends on how they are imagined, governed and contested. Hope does not replace sound policy or technical expertise; but rather it provides the civic and institutional capacity to align knowledge, agency and governance so that urban systems are intentionally designed to generate health.

Realising this shift requires deliberate institutionalisation; with new and reconfigured institutions designed explicitly to cultivate and sustain hope as a public (health) capacity. Such institutions would be well placed to serve as hope infrastructure: scaffolding the imagination, knowledge production and collective action necessary to ensure that urban infrastructure delivers health and climate resilience for all.
